# Voriconazole successfully treats intracranial *Trichosporon asahii* infection in an immunocompetent patient: a rare case report and literature review

**DOI:** 10.3389/fmed.2025.1560016

**Published:** 2025-03-20

**Authors:** Cuilin He, Danjie Zhao, Xiwen Wang, Hua Wang, Lingmei Huang, Maozhu Liu, Enqiang Chen

**Affiliations:** ^1^Department of Pharmacy, The First People’s Hospital of Shuangliu District (West China Airport Hospital of Sichuan University), Chengdu, China; ^2^Department of Clinical Laboratory, The First People’s Hospital of Shuangliu District (West China Airport Hospital of Sichuan University), Chengdu, China; ^3^Department of Neurosurgery, The First People’s Hospital of Shuangliu District (West China Airport Hospital of Sichuan University), Chengdu, China; ^4^Center of Infectious Diseases, West China Hospital, Sichuan University, Chengdu, China

**Keywords:** intracranial infection, *Trichosporon asahii*, immunocompetent patient, antifungal treatment, treatment efficacy

## Abstract

*Trichosporon asahii* (*T. asahii*) has been increasingly recognized as the causative pathogen of invasive fungal infection, whereas intracranial infection caused by *T. asahii* are extremely scarce. Here, we report a rare case of intracranial *T. asahii* infection in an immunocompetent woman from China. She was hospitalized for obstructive hydrocephalus and experienced two brain surgeries. One week after the second surgery, the patient developed fever, vomiting, and elevated infection-related indicators. Cerebrospinal fluid (CSF) cultures yielded *T. asahii* and its morphology was demonstrated by Gram staining. The patient initially received empiric antifungal therapy with voriconazole (VCZ), and the subsequent drug sensitivity results supported the continuation of this therapy. Finally, 15 days of VCZ administration successfully achieved satisfactory therapeutic effects. This case highlights that *T. asahii* has emerged as an infectious cause of intracranial fungal infection in immunocompetent people. Early recognition and adequate antifungal treatment are paramount to ensure a favorable prognosis.

## 1 Introduction

Invasive fungal infection is a serious life-threatening disease in humans, and their attributable mortality rates continue to rise with the expanding use of immunosuppressive agents (1). *Trichosporon* species are opportunistic fungal pathogens that are widespread in nature and colonize human skin, mucosal surfaces, and gastrointestinal tract, causing fungal infection not only in immunocompromised hosts but also in immunocompetent hosts (2). Noteworthy, *Trichosporon asahii* (*T. asahii*), the most common etiologic agent of *Trichosporon* species, is of increasing concern due to its ability to cause fatal infection with high mortality (3, 4). Until now, numerous studies have emphasized that *T. asahii* infection occurs in multiple organs, resulting in fungemia, urinary tract infection, subcutaneous mycoses, and keratitis, imposing a heavy infection burden on patients (4–7). However, *T. asahii* has rarely been considered the causative pathogen of intracranial infection. The first brain trichosporonosis was reported in 1970, and the patient died 4 weeks after hospital admission (8). To date, only five available cases of CNS *T. asahii* infection have been published, and the current treatment experience is still insufficient (9–13).

*T. asahii* exhibits high resistance to certain antifungal drugs, a characteristic that poses a serious challenge to clinical management, particularly for patients receiving long-term antibiotic therapy (4, 14). It is reported that *T. asahii* has intrinsic resistance to echinocandins and therefore echinocandins are not advocated for treating *T. asahii* infections (2). Triazoles can successfully treat *T. asahii* infection in different tissues and organs, with VCZ therapy resulting in higher survival rates than other antifungal drugs (1, 6, 15, 16). As for intracranial *T. asahii* infection, only two cases have been reported in which patients ultimately cured after receiving antifungal therapy (11, 13). One was treated with amphotericin B (AMB) followed by itraconazole (ITC) (11). The other was initially treated with AMB + voriconazole (VCZ) and then continued with VCZ (13). These existing studies provide limited therapeutic experiences in immunocompromised patients, but not in immunocompetent populations. Considering the potentially lethal consequences of brain infection, we believe intracranial *T. asahii* infection deserves more attention and more clinical practice is required to optimize the treatment strategies. In this report, we present a case of intracranial *T. asahii* infection successfully treated with VCZ in an immunocompetent patient who had undergone brain surgeries. We hope this study can provide references for accurate recognition and rational treatment of *T. asahii* infection in clinical practice.

## 2 Case presentation

In July 23, 2024, a 66-year-old woman was admitted to our hospital with a 2-month history of obstructive hydrocephalus. Before this patient arrived at our hospital, she was diagnosed with a ruptured and hemorrhaged left posterior inferior cerebellar artery aneurysm and underwent intracranial surgery at another hospital. Postoperatively, the patient developed obstructive hydrocephalus and fever on June 4, 2024. Carbapenem-resistant *Acinetobacter baumannii* was later identified in blood and CSF cultures on June 11, 2024. After about 2 months of successive treatment with different antibiotics such as meropenem, vancomycin, polymyxin, and amikacin, her infection was eventually well controlled. Afterward, she presented to our hospital for further control of obstructive hydrocephalus.

**FIGURE 1:**
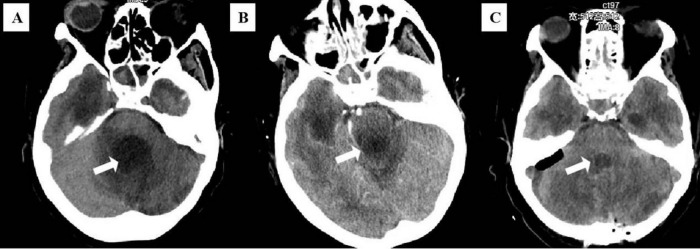
**(A)** Brain computed tomography (CT) images at the initial diagnosis. **(B)** Brain CT before the second cranial surgery on 4th day. **(C)** Brain CT after the second cranial surgery on 9th day. White arrows indicate the lesion in the fourth ventricle.

Supplementary Figure 1 illustrates the timeline of the patient’s disease progression during hospitalization. On admission, she presented with a blood pressure of 119/71 mmHg, heart rate of 66 beats/minute, respiratory rate of 19 breaths/minute, body temperature of 35.0°C, and oxygen saturation in arterial blood (SaO_2_) of 95.3%. Physical examination revealed mild coma with a Glasgow Coma Scale score of 4, decreased pupillary light reflexes in both eyes, neck stiffness, and a positive Babinski sign. The infection markers such as white blood cell count (WBC) and percentage of neutrophilic granulocyte (NEUT%) were within the normal limits. CSF was abnormal which showed glucose 5.36 mmol/L, protein content 2232.5 mg/dL, and WBC 84 × 10^6^/L (Table 1). Computed tomography (CT) of the brain indicated dilation and communicating hydrocephalus in the bilateral ventricles, the third and fourth ventricles (Figure 1A). The primary diagnosis was subsequently confirmed as obstructive hydrocephalus.

**TABLE 1:** Results of laboratory testing after admission.

Indicator	Reference ranges	Day1	Day10	Day13	Day14	Day15	Day16	Day21	Day22	Day24	Day29
T_max_ (°C)	–	37.1	36.7	37.9	36.6	38.2	38.3	38.0	37.8	37.2	37.3
WBC (×10^9^/L)	3.5–9.5	7.66	12.19	7.73	8.98	11.38	11.29	5.36	–	5.68	3.25
NEUT% (%)	40–75	73.89	85.97	79.17	81.63	83.92	82.99	78.92	–	71.3	54.77
CRP (mg/L)	0–10	86.03	–	–	–	–	–	–	–	–	–
PCT (ng/mL)	0–0.046	0.18	0.66	–	–	0.26	–	–	–	–	–
ALT (U/L)	7–40	110.2	38.4	37.9	36.6	38.2	38.2	43.8	–	29.2	20.3
AST (U/L)	13–35	113.1	46.9	24.3	25.2	28.2	30.1	50.6	–	34.5	32.4
GGT (U/L)	7–45	26.2	24	–	–	–	30.2	54.9		49	60.8
ALP (U/L)	50–135	86.1	70.8	–	–	–	130.6	119.2		107.7	100.4
Cr (μmol/L)	41–81	63.5	48.8	39.4	41.2	35.6	43	38.3	–	33.3	37
eGFR (mL/min)	56–122	87.5	97.6	104.7	103.3	108.3	101.8	105.7		110.7	106.9
CSF Glu (mmol/L)	2.2–3.9	5.36	4.6	3.35	–	3.96	–	–	–	–	–
CSF Pro (mg/dL)	15–45	2232.5	186.1	128	–	172.2	–	–	–	–	–
CSF WBC (×10^6^/L)	–	84	295	172	–	323	–	–	–	–	–
SO_2_ (%)	91.9–99	95.3%	99%	99%	99%	≥95%	≥95%	≥95%	≥95%	≥95%	≥95%

T_max_, body temperature peak, WBC, white blood cell; NEUT%, percentage of neutrophilic granulocyte; CRP, C-reactive protein; PCT, procalcitonin; ALT, alanine aminotransferase; AST, aspartate aminotransferase; GGT, γ-glutamyl transpeptidase; ALP, alkaline phosphatase; Cr, serum creatinine; eGFR, estimated glomerular filtration rate; CSF, cerebrospinal fluid; Glu, glucose; Pro, proteins; SO_2_, oxygen saturation.

**FIGURE 2:**
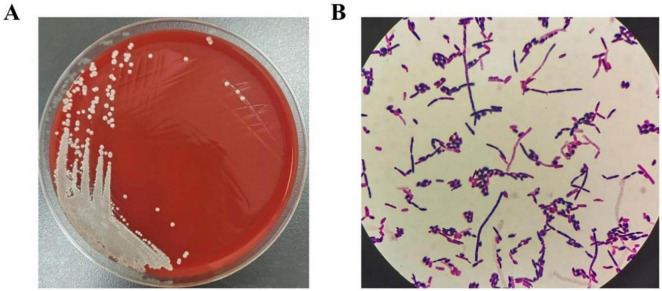
**(A)** *T. asahii* in CSF appears as white yeast-like fungus after 24 h of incubation on blood agar plates. **(B)** Microscopy of isolates (Gram staining) showing *T. asahii* septate hyphae with arthrospores.

On the 5th hospital day, the patient experienced projectile vomiting with a heart rate dropping to 40 beats/minute, which was considered to be a consequence of exaggerated obstructive hydrocephalus. She immediately underwent surgical treatment with brain ventricle puncture and drainage. After surgery, the vomiting improved, but pathological breathing developed and her heart rate decreased to 35–60 beats/minute. A brain CT scan showed obvious enlargement of the fourth ventricle with compression of the medulla oblongata (Figure 1B). As a result, a second surgery (brain ventricle puncture and drainage, fourth ventricular lesion resection, and suboccipital decompression) was performed on the 8th day of hospitalization. Postoperatively, her symptoms gradually subsided, and CT demonstrated evidence of improvement in ventricular dilation and effusion (Figure 1C). On the 15th day of admission, she suffered from vomiting and intermittent fever, with a maximum temperature of 38.2°C. As listed in Table 1, laboratory tests demonstrated a significantly elevated WBC (11.38 × 10^9^/L) and NEUT% (83.92%). CSF analysis revealed glucose of 3.96 mmol/L, protein of 172.2 mg/dL, and WBC of 323 × 10^6^/L, all of which were abnormal. Considering that the obstructive hydrocephalus had significantly improved and that prolonged drain placement may contribute to infection, the ventricular drainage device was removed. Subsequently, her CSF, sputum, and blood were collected for culture. Considering no pathogenic microorganisms were detected in the sputum and blood cultures, and neither the chest CT scan nor the clinical presentation showed signs of lung infection, lung and blood infections were excluded. However, the CSF cultures were positive after 24 h of incubation, and isolates from CSF cultures showed septate hyphae with arthrospores by Gram staining (Figure 2). The species in the CSF cultures was then identified as *T. asahii*, and the diagnosis of intracranial *T. asahii* infection was established. On the 17th hospital day, voriconazole (0.4 g/twice daily for two doses, then 0.2g/twice daily for 2 weeks, intravenously) was empirically administered to combat *T. asahii* invasion. Shortly after, antifungal susceptibility testing proved that voriconazole exhibited potent activity against *T. asahii* with a MIC of 0.125 μg/mL (Table 2). After 5 days of voriconazole treatment, the patient was afebrile and her serum WBC and NEUT% returned to normal (Table 1). Consequently, the patient completed 15 days of voriconazole monotherapy and was discharged on hospital day 55 in stable condition and without recurrent infection.

**TABLE 2:** The susceptibility results of *T. asahii* isolated from CSF.

Antifungal agent	MIC (μg/mL)
5-Flucytosine	≥16
Amphotericin B	≤0.5
Fluconazole	2
Itraconazole	0.25
Voriconazole	0.125

MIC, minimal inhibitory concentration.

## 3 Pathogen identification and antifungal susceptibility testing

Cerebrospinal fluid (CSF) was inoculated onto blood agar plates for 24 h at 35°C under aerobic conditions (Figure 2A). Yeast-like fungi were isolated from the CSF cultures and showed septate hyphae with arthrospores by Gram staining under microscopy (Figure 2B). The fungus was further identified by Matrix-Assisted Laser Desorption/Ionization Time-of-Flight (MALDI-TOF) technology (Zybio, China, EXS 2600). According to the interpretation criteria, identification scores <1.7 indicate unreliable identification, 1.7–2.0 indicate genus-level identification, and ≥2.0 indicate species-level identification. The isolate in our case was identified at the species-level with an acceptable score of 2.06, thus the results provide a reliable *T. asahii* identification. Antifungal drug susceptibility analysis was performed by broth microdilution method with VITEK-2 Compact fully automated microbial susceptibility analysis system (France bioMérieux). The minimum inhibitory concentrations (MICs) of the antifungal drugs are listed in Table 2.

## 4 Review and discussion

Invasive infection induced by *T. asahii* is considered extremely fatal and difficult to treat, as it usually progresses rapidly, with 78% of patients dying even after receiving antifungal therapy (2, 17). Therefore, delayed diagnosis and inadequate treatment indeed aggravate patient suffering and increase mortality rates. Despite numerous reports indicating that *T. asahii* is responsible for infections in multiple tissues and organs, it has rarely been reported as the etiological agent of intracranial infection (18, 19). We believe its invasiveness and pathogenicity in the CNS cannot be ignored. Supplementary Table 1 summarizes the five existing cases of CNS *T. asahii* infection, among which only two reported that the patients were ultimately recovered following antifungal therapy. The first case was reported in 2007 involving a non-immunocompromised host who partially responded to fluconazole therapy but finally lost to follow-up (9). In 2011, a second case of *Trichosporon* meningitis and cerebral abscess was described in a burn patient who succumbed to multiorgan dysfunction prior to receiving appropriate antifungal therapy (10). Subsequently, the first case of successful treatment of CNS *T. asahii* infection was published, in which a patient undergoing immunosuppressive therapy for autoimmune hepatitis was diagnosed with a *Trichosporon* brain abscess, which resolved after treatment with AMB and ITC (11). In 2015, an immunocompetent patient presenting with chronic meningo-ventriculitis and intra ventricular fungal ball caused by *T. asahii* was reported (12). This patient received empirical treatment with liposomal amphotericin B but unfortunately died rapidly from cardiac arrest shortly thereafter. Soon after, another study detailed an allogeneic hematopoietic stem cell transplant (allo-HSCT) recipient suffering from invasive CNS trichosporonosis who achieved recovered after administering VCZ (13). It is noteworthy that both cured patients were immunosuppressed, and considering that there are no references on the successful management of intracranial *T. asahii* infection in immunocompetent populations, we report a rare intracranial *T. asahii* infection in a 66-year-old immunocompetent female in China.

To date, the exact mechanism by which *T. asahii* infects humans is not fully understood. The available data suggest that the infection mechanisms of *T. asahii* may be related to its adherence proteins binding to host proteins (e.g., serum albumin, α-1antitrypsin, and vitronectin) (20). Nowadays, increasing studies have discovered that *T. asahii* primarily affects immunocompromised patients and rarely infects individuals with normal immunity (1, 21, 22). Interestingly, the patient in our case was immunocompetent. It was found that other factors besides immune status can also increase the risk of *T. asahii* infection, especially antibiotic use and invasive medical devices (23). Among them, it was demonstrated that *T. asahii* can escape the immune response by adhering to implanted devices to form biofilms, which can facilitate its invasion (2). We speculate that this feature may contribute to the *T. asahii* infection in immunocompetent patients. Consistent with these findings, our patient had an underlying history of prolonged exposure to antibiotics, brain surgeries, and drainage device placement, all of which are recognized as existing risk factors contributing to intracranial *T. asahii* infection. Furthermore, *T. asahii* is a commensal of human skin and can infect humans from colonized skin via catheters or central lines (1, 24). We suspect that *T. asahii* in this case was transferred from human skin to the brain through ventricular drainage devices.

Notably, *T. asahii* is inherently resistant to multiple antifungal drugs, and its limited therapeutic options have become a matter of concern. To date, there are no specific therapeutic recommendations for *T. asahii* infection as the MIC breakpoints have not been defined and convincing evidence from randomized clinical trials is lacking (25). Since echinocandins exhibit no antifungal activities against *T. asahii*, they are not advocated for treating *T. asahii* infection (25, 26). The 2021 global guideline for the diagnosis and management of rare yeast infections recommends VCZ as a first-line agent for initial therapy against *T. asahii* (25). However, this recommendation is primarily based on animal studies, *in vitro* experiments, and case reports, and the optimal antifungal therapy for *T. asahii* infection remains uncertain. An in-depth study comparing the effects of various triazoles on clinically isolated *T. asahii* showed that VCZ had the highest fungistatic activity, followed by itraconazole, posaconazole, and isavuconazole, which had equal activity against *T. asahii*, whereas fluconazole showed the lowest antifungal activity (26). From the above, we can see the remarkable efficacy of VCZ on *T. asahii* infection. When compared to polyenes, guidelines suggest that VCZ is superior to AMB because *T. asahii* is usually resistant to AMB (MICs ≥ 2 μg/mL) (25, 27). Furthermore, some case reports have demonstrated that switching to VCZ resulted in satisfactory patient outcomes when AMB-based regimens failed to control *T. asahii* infection (17, 28). Based on this information, VCZ appears to be a better therapeutic choice for invasive trichosporiasis than AMB. Moreover, combination therapy with VCZ and AMB was also utilized in some studies, but the results were not superior to monotherapy (23). Referring to the 2021 guidelines, azole-polyene combinations are recommended as salvage therapy since it has no advantage as initial therapy (25). Therefore, a combination regimen was not adopted for the initial treatment of our patient. It is worth noting that although there is sufficient evidence that azoles are the best first-line antifungal therapy agents for *T. asahii* infection, some findings indicate that long-term use of azoles therapy may lead to mutations in the *ERG11* gene, resulting in the emergence of azole-resistant isolates (3).

In our case, the patient was empirically started on VCZ according to accessible guidelines and literature. Subsequently, the results of antifungal susceptibility testing revealed that the isolate had low MICs for VCZ (MIC = 0.125 μg/mL), AMB (MIC ≤ 0.5 μg/mL), itraconazole (MIC = 0.25 μg/mL), and fluconazole (MIC = 2 μg/mL). Combining previous studies and our data, we consider that VCZ monotherapy may be the preferred regimen for our patient, and the patient continued administering VCZ 400 mg/day for an adequate duration. After 15 days of VCZ monotherapy, the patient’s infection symptoms resolved completely without serious complications or recurrence until discharge. Indeed, the duration of antifungal therapy required for recovery in our patients was relatively short compared to other reported patients with CNS *T. asahii* infection (11, 13). We hypothesize that one possible explanation is that our patient had normal immune function that facilitates microbial pathogen clearance. On the other hand, we provided rapid diagnosis and effective treatment to control the infection before it worsened successfully. Based on these factors, her intracranial *T. asahii* infection was eventually cured rapidly.

## 5 Conclusion

We describe a case of intracranial *T. asahii* infection in an immunocompetent patient who was ultimately cured by VCZ. It is worth reflecting that timely diagnosis and appropriate clinical treatment are key to a favorable prognosis. Initiation of azole antifungal therapy, particularly VCZ, may be an optimal choice to control intracranial *T. asahii* infection. Moreover, clinicians should remain vigilant that *T. asahii* infection is not restricted only to immunosuppressed populations. Long-term antibiotic use, surgical operation, or drainage device placement may increase the risk of *T. asahii* infection in immunocompetent individuals, and close monitoring of these populations can prevent the occurrence of *T. asahii* infection.

## Data Availability

The original contributions presented in this study are included in this article/Supplementary material, further inquiries can be directed to the corresponding authors.
