# Supplementary material for: Voriconazole successfully treats intracranial Trichosporon asahii infection in an immunocompetent patient: a rare case report and literature review
Source: Front Med (Lausanne). 2025 Mar 20;12:1560016. doi: 10.3389/fmed.2025.1560016 (PMC11969222; doi:10.3389/fmed.2025.1560016)
Supplement: Supplementary file 1 [file Supplementary_file_1.doc]

***Supplementary Material***

**
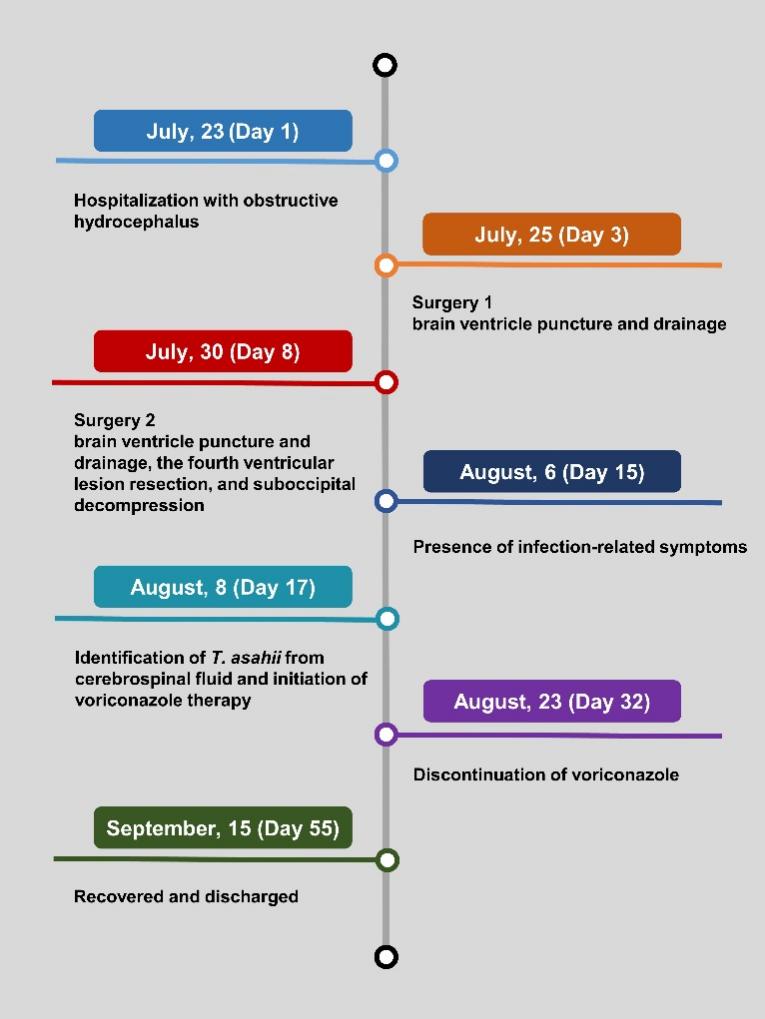
**

**Figure S1.** Timeline of the case progression of the patient from admission to discharge.

**Table S1.** Reported cases of CNS *T. asahii* infection.

| **Reference** | **Country** | **Gender/age** | **Diagnosis** | **Risk factors** | **Sample** | **Treatment** | **Outcome** |
| --- | --- | --- | --- | --- | --- | --- | --- |
| Thien SY et al. 2016 (1) | Singapore | F/50 | Disseminated trichosporonosis | Aplastic anemia and allo-HSCT | CSF and blood | AMB + VOR for 21 months, followed by VOR for life long | Survived |
| Kumar A et al. 2015 (2) | Burundi | M/18 | Meningo-ventriculitis and intraventricular fungal ball | Unknown | Brain tissue and CSF | AMB for 10 days | Died |
| Basiri K et al. 2012 (3) | Iran | M/34 | Brain abscess | Immunosuppressant use | Brain abscess | AMB for 3 weeks, followed by ITC for 1 year | Survived |
| Heslop OD et al (4) | India | F/44 | Meningitis and cerebral abscess | Diabetic, 50% burns, and broad spectrum antibiotic use | Meninges, brain abscess, facial wounds, and sputum | None | Died |
| Rastogi VL et al. 2007 (5) | India | M/18 | Meningoencephalitis and pneumonia | Diabetic | CSF | FLU for 13 days | Loss to follow-up |

*CNS* central nervous system, *CSF* cerebrospinal fluid, *allo-HSCT* allogeneic hematopoietic stem cell transplantation, *AMB* amphotericin B, *VOR* voriconazole, *ITC* itraconazole, *FLU* fluconazole, *F* female, *M* mal

1. Thien SY, Chung SJ, Tan AL, Hwang WY, Tan BH, Tan TT. Recurrent trichosporonosis with central nervous system involvement in an allogeneic hematopoietic stem cell transplant recipient. Transpl Infect Dis. 2016;18(5):768-72.

2. Kumar A, Udayakumaran S, Babu R, Rajamma BM, Prakash A, Panikar D, et al. Trichosporon asahii infection presenting as chronic meningo-ventriculitis and intra ventricular fungal ball: a case report and literature review. Mycoses. 2015;58(2):99-103.

3. Basiri K, Meidani M, Rezaie F, Soheilnader S, Fatehi F. A rare case of Trichosporon brain abscess, successfully treated with surgical excision and antifungal agents. Neurol Neurochir Pol. 2012;46(1):92-5.

4. Heslop OD, Nyi Nyi MP, Abbott SP, Rainford LE, Castle DM, Coard KC. Disseminated trichosporonosis in a burn patient: meningitis and cerebral abscess due to Trichosporon asahii. J Clin Microbiol. 2011;49(12):4405-8.

5. Rastogi VL, Nirwan PS. Invasive trichosporonosis due to Trichosporon asahii in a non-immunocompromised host: a rare case report. Indian J Med Microbiol. 2007;25(1):59-61.
